# Complementary and Alternative Medicine and Cardiovascular Disease: An Evidence-Based Review

**DOI:** 10.1155/2013/672097

**Published:** 2013-04-22

**Authors:** Matthew J. Rabito, Alan David Kaye

**Affiliations:** ^1^Department of Anesthesiology, Louisiana State University Health Sciences Center, School of Medicine, 1542 Tulane Avenue, Room 656, New Orleans, LA 70112, USA; ^2^Department of Pharmacology, Louisiana State University Health Sciences Center, School of Medicine, 1542 Tulane Avenue, Room 656, New Orleans, LA 70112, USA

## Abstract

Complementary and alternative medicine (CAM) plays a significant role in many aspects of healthcare worldwide, including cardiovascular disease (CVD). This review describes some of the challenges of CAM in terms of scientific research. Biologically-based therapies, mind-body therapies, manipulative and body-based therapies, whole medical systems, and energy medicine are reviewed in detail with regard to cardiovascular risk factors and mediation or modulation of cardiovascular disease pathogenesis. CAM use among patients with CVD is prevalent and in many instances provides positive and significant effects, with biologically-based and mind-body therapies being the most commonly used treatment modalities. More rigorous research to determine the precise physiologic effects and long-term benefits on cardiovascular morbidity and mortality with CAM usage, as well as more open lines of communication between patients and physicians regarding CAM use, is essential when determining optimal treatment plans.

## 1. Introduction 

The National Center for Complementary and Alternative Medicine (NCCAM) defines complementary and alternative medicine (CAM) as “a group of diverse medical and health care systems, practices, and products that are not generally considered part of conventional medicine” [1]. Complementary medicine is used along with conventional medicine, whereas alternative medicine is used in place of conventional medicine. In the 2007 National Health Interview Survey (NHIS), approximately 38% of USA adults and 12% of children reported using CAM in the past 12 months, and lifetime prevalence of CAM use in the United States and worldwide has increased steadily since 1950 [2, 3]. A systematic search of the existing literature found that the prevalence of CAM use ranges between 5% and 74.8% [3]. The NHIS report also noted that 83,000,000 adults spent $33.9 billion out of pocket on CAM, constituting 11.2% of total out-of-pocket health care expenditures and approximately 1.5% of total health care expenditures [2]. 

 The 2007 NHIS report demonstrated that the top five most frequently used CAM therapies (excluding prayer) were natural products, such as fish oil/omega 3, glucosamine, echinacea, and flaxseed (17.7%), deep breathing (12.7%), meditation (9.4%), chiropractic and osteopathic (8.6%), and massage (8.3%), followed by yoga, diet-based therapies, progressive relaxation, guided imagery, and homeopathic treatment [2]. Frass et al. report that the most used therapies are chiropractic manipulation, followed by phytotherapy/herbal medicine, massage, and homeopathy [3]. The conditions for which CAM is most frequently used according to the 2007 NHIS include back pain, neck pain, joint pain, arthritis, and anxiety, while Frass et al. report the top five conditions to be back pain/neck problems, depression, insomnia/trouble sleeping, severe headache/migraine, and stomach/intestinal illness [2, 3]. CAM use among adults is greatest among women and those of middle age who are better educated and have higher incomes [2, 3].

 The scientific study of CAM poses several unique challenges that must be taken into consideration in order to adequately produce and assess evidence-based data. Many forms of CAM are elements of broader healing systems based on unique theoretical constructs and systems of analysis, rather than stand-alone treatments—isolating a particular therapy (i.e., acupuncture) from its broader discipline (i.e., Chinese medicine) may not do it justice. There is the potential for disparity in disciplines: Chinese, Korean, and Japanese acupuncture styles are different, and adjunct therapies such as manual or electrical needle stimulation and use of herbal preparations orally or through moxibustion add to the disparity. Another potential confounder is that traditional outcome measures may not capture the full effect of treatment, since many such therapies do not have well-recognized and understood physiologic mechanisms of action (i.e., *qi* or *chi* in Chinese medicine). The placebo effect must also be considered. Because many of the popular CAM therapies are in fact physical methods (i.e., massage or acupuncture) of treatment, it is difficult to formulate a placebo that is both inert and indistinguishable from the real treatment. Additionally, the lack of a uniform definition of CAM and the huge diversity of the different methods, therapies, and dogmas of CAM make the studies difficult to compare [3]. Enthusiasm, individuality, and the specific nature of the doctor-patient relationships play a role. Finally, the quality of these trials is frequently lacking in that many have small sample sizes and are not prospective, randomized, rigorously conducted, placebo-controlled studies, and they often have poor methodological characteristics and high incidences of bias.

 Cardiovascular disease (CVD) is the leading cause of mortality in the United States for both men and women [4]. Approximately 600,000 people die of heart disease in the United States every year, representing one in every four deaths [4]. The most common form of CVD is coronary heart disease, which kills more than 358,000 people and costs the United States $108.9 billion each year [4]. Risk factors for CVD include hypertension, high LDL-cholesterol, smoking, diabetes, overweight and obesity, poor diet, physical inactivity, and excessive alcohol use [4]. Despite growing interest in CAM for cardiovascular health, few data are available regarding patterns of use of CAM for cardiovascular disease in the United States [5]. One study used the 2002 NHIS to analyze data on CAM use among patients with CVD and found that 36% of patients with CVD had used CAM (excluding prayer) in the previous 12 months [5]. Herbal products (echinacea, garlic, ginseng, ginkgo biloba, and glucosamine) and mind-body therapies (deep-breathing exercises and meditation) were used by 18% and 17% of patients, respectively, and constituted the most commonly used therapies [5]. Overall, CAM use in this patient population mirrored CAM use in the general population, with the most common reasons for use being musculoskeletal complaints, anxiety/depression, and stress/emotional health and wellness [5]. According to this study, fewer respondents (10%) used CAM specifically for their cardiovascular conditions (5% for hypertension, 2% for coronary disease, 3% for vascular insufficiency, <1% for heart failure or stroke) [5]. 

Another study with an international basis of patient information found that the prevalence of CAM use among people with CVD ranged from 4% to 61% (biologically-based therapies 22%–68%, herbal medicine 2%–46%, vitamins/minerals/dietary supplements 3%–54%, and mind-body therapies 2–57%) [6]. This review found that the use of CAM specifically to treat CVD ranged from 7% to 82%, depending on the study [6]. Physician awareness of their patients' CAM therapy use ranged from 8% to 65% secondary to fear of physician disapproval and lack of inquiry on the subject by the physician [6]. Some reasons for CAM use cited by patients include that CAM was thought to be of greater benefit than conventional medications (15%), adverse drug reactions to conventional therapy (59%), and overall well-being and promotion of good health [6].

## 2. CAM Effects on the Cardiovascular System and CVD Risk Factors

 CAM may be broadly divided into 5 separate categories: biologically-based therapies, mind-body therapies, manipulative and body-based therapies, whole medical systems, and energy medicine [3].

### 2.1. Biologically-Based Therapies

 The biologically-based therapies include aromatherapy, chelation therapy, diet-based therapies, folk medicine, iridology, megavitamin therapy, neural therapy, and phytotherapy/herbal medicine [3]. A number of these therapies have purported cardiovascular effects, but most research on these products is either inconclusive, conflicting, or shows no benefit for their use. It is equally important for the advancement of the legitimate and rigorous study of CAM to report negative as well as positive results, and it illustrates the need for studies of higher quality in this area.

Marine-derived omega-3 polyunsaturated fatty acids (fish oil) are often touted as being preventative of major cardiovascular adverse outcomes by the postulated mechanisms of lowering triglyceride levels (for which they are approved by the United States Food and Drug Administration (FDA)), preventing arrhythmias, decreasing platelet aggregation, or lowering blood pressure [7]. And while experts agree that fish rich in omega-3 fatty acids should be included in a heart-healthy diet, there is no evidence that omega-3 fatty acids in supplement form protect against heart disease [1]. A recent meta-analysis found that omega-3 polyunsaturated fatty acid supplementation was not associated with a lower risk of all-cause mortality, cardiac death, sudden death, myocardial infarction, or stroke based on relative and absolute measures of association [7].

 Garlic is used most frequently as a dietary supplement for treatment of hyperlipidemia, heart disease, and hypertension. A well-conducted, randomized trial demonstrated that there was no significant difference in LDL-cholesterol, HDL-cholesterol, triglycerides, or total cholesterol-HDL ratio after six months of treatment with three preparations of garlic versus placebo [8]. There is evidence that garlic is associated with blood pressure reductions in patients with elevated systolic blood pressures (10–12 mm Hg systolic, 6–9 mm Hg diastolic), but not in normotensive patients [9–11]. However, there is insufficient evidence to determine whether garlic provides a therapeutic advantage versus placebo in terms of reducing the risk of cardiovascular morbidity and mortality [9].

 There is some evidence that ginseng has a plethora of cardiovascular benefits, including cardioprotection, antihypertensive effects, and attenuation of myocardial hypertrophy and heart failure [12]. However, a randomized, double-blind, placebo-controlled study demonstrated that Korean red ginseng had no significant effect on blood pressure, lipid profile, oxidized low density lipoprotein, fasting blood glucose, or arterial stiffness in subjects with metabolic syndrome [13]. A systematic review and meta-analysis also failed to demonstrate superiority of red ginseng over placebo in regard to effectiveness for type 2 diabetes mellitus [14].

 Ginkgo biloba is purported to have cardioprotective effects by several studies through its antioxidant, antiplatelet, antithrombotic, vasodilatory, and antihypertensive properties [15]. A double-blind, placebo-controlled, randomized clinical trial determined, however, that the herb does not reduce blood pressure or the incidence of hypertension in elderly men and women [15]. The trial also noted that there was no evidence that ginkgo biloba reduced total or CVD mortality or CVD events; there were, however, more peripheral vascular disease events in the placebo arm, suggesting that the herb may reduce the risk of developing peripheral arterial disease [16]. 

 Hawthorn leaf and flower extracts are advocated as an oral treatment option for patients with chronic heart failure; in fact, the German Commission E approved the use of hawthorn extracts in patients with heart failure graded stage II [17]. The results of a Cochrane review suggest that there is a significant benefit in symptom control and physiologic outcomes from hawthorn extract as a treatment adjunct for chronic heart failure [17]. For the physiologic outcome of maximal workload, treatment with hawthorn extract was more beneficial than placebo [17]. Hawthorn extract increased exercise tolerance, beneficially decreased cardiac oxygen consumption, and improved symptoms such as shortness of breath and fatigue as compared with placebo [17]. However, no data on relevant mortality and morbidity were reported [17]. The SPICE trial, a large, randomized, placebo-controlled, double-blind study, specifically looked at morbidity and mortality as endpoints [18, 19]. The study concluded that the primary endpoints—reductions in cardiac death, nonfatal myocardial infarction, and hospitalization due to progressive heart failure—were not achieved [19]. The SPICE trial also found that the deaths of a sudden cardiac cause, deaths due to progressive heart failure, and fatal myocardial infarctions were all lower in the treatment group; these figures, however, did not reach statistical significance [19]. Finally, the study suggested that treatment with hawthorn may reduce sudden cardiac deaths specifically in patients with left ventricular ejection fractions between 25% and 35% [19].

 A meta-analysis determined that overall, flaxseed supplementation was associated with a decrease in blood total and LDL-cholesterol concentrations but did not significantly affect HDL-cholesterol and triglycerides [20]. The study reported that whole flaxseed interventions resulted in significant reductions in total and LDL-cholesterol, while flaxseed oil did not [20]. Flaxseed contains a large amount of fiber, and dietary soluble fiber has been shown to have cholesterol-lowering effects [20]. Flaxseed is also a rich source of dietary lignans. Purified lignans have been shown to reduce cholesterol in animal studies, but human data are limited [20]. Importantly, the beneficial effects of flaxseed were observed only among those with relatively high initial cholesterol concentrations and were more apparent in females (particularly postmenopausal females) [20]. A multitude of other cardiovascular benefits have been proposed for flaxseed due to its high alpha-linolenic acid content [21–23], but not enough reliable data are available to determine whether flaxseed is effective for heart disease in humans.

 Antioxidants, which include anthocyanins, beta-carotene, catechins, coenzyme Q10, flavonoids, lipoic acid, lutein, lycopene, selenium, and vitamins C and E, have shown promising results in laboratory and observational studies; however, systematic reviews of the literature and large, randomized, controlled trials have generally found no beneficial effects of antioxidant supplements for primary or secondary prevention. In fact, vitamin A, beta-carotene, and vitamin E may actually increase mortality [24]. The Physicians Health Study II concluded that neither vitamin E nor vitamin C reduced the risk of major cardiovascular events (nonfatal myocardial infarction, nonfatal stroke, and CVD death) or had a significant effect on total mortality [25]. The Women's Health Study concluded that, overall, vitamin E did not reduce the risk of death or major cardiovascular events (myocardial infarction, stroke, or death) in almost 40,000 healthy women; however, there was a significant 24% reduction in the secondary endpoint of cardiovascular deaths and a significant 26% reduction in major cardiovascular events among a subgroup of women aged at least 65 years [26]. The Women's Antioxidant Cardiovascular Study found that there were no overall effects of vitamins C, E, or beta-carotene on cardiovascular events among women at high risk for CVD [27]. Possible reasons for the “disconnect” between findings of laboratory and observational studies and results of clinical trials, according to one article, may be that trials are entirely too short to reverse the results of decades of oxidative stress contributing to atherosclerosis or that the antioxidants selected for study were chosen for their easy availability rather than proven efficacy (vitamin E) [28].

 Red yeast rice contains monacolin K, which has the same chemical structure as lovastatin, an inhibitor of HMG-CoA reductase [29]. Monacolin K in substantial amounts lowers blood levels of total cholesterol and LDL-cholesterol [29–31]. One study reported, however, that there is marked variability of monacolin levels in commercial red yeast rice products, and several products had elevated levels of citrinin, a potentially nephrotoxic mycotoxin [32]. Products with very little monacolin K may have little to no effect on blood cholesterol levels. Although red yeast rice has been marketed to patients intolerant of statin drugs due to those drugs' side effects, there have been case reports of myopathy and rhabdomyolysis associated with red yeast rice [29]. In 1998, the FDA ruled that a red yeast rice product that contained a substantial amount of monacolin K was no longer a dietary supplement but an unapproved new drug and that marketing the product as a dietary supplement would be illegal [1]. Despite FDA action, some products tested as recently as 2011 have been found to contain substantial amounts of monacolin K [1]. Consumers therefore have no way of knowing how much monacolin K is present in most red yeast rice products or whether a particular product is safe, effective, or legal [1].

 Soy protein and isoflavones (phytoestrogens) have gained attention for their potential role in improving risk factors for CVD [33]. An American Heart Association scientific advisory concluded that isolated soy protein with isoflavones, as compared with milk or other proteins, decreased LDL-cholesterol concentrations by an average amount of approximately 3% when about half of the usual total daily protein intake was soy protein [33]. No significant effects on HDL-cholesterol, triglycerides, lipoproteins, or blood pressure were evident [33]. Earlier research indicating that soy protein has clinically important favorable effects has not been confirmed by the meager evidence from clinical trials [33].

 L-carnitine is FDA approved for replacement therapy in primary (i.e., inborn errors of metabolism) and secondary (i.e., secondary to hemodialysis) L-carnitine deficiencies [34]. Many clinical trials have suggested acetyl-L-carnitine (ALC) and propionyl-L-carnitine (PLC), two naturally occurring carnitine derivates, as potential strategies in the management of peripheral arterial disease (PAD), heart and cerebral ischemia, and congestive heart failure [35]. The beneficial effects of PLC on PAD, particularly in alleviating intermittent claudication, have been widely studied. It is generally agreed upon that PLC is able to improve exercise tolerance in terms of increasing the maximum walking distance in patients suffering from intermittent claudication as well as to improve most measures of quality of life (overall physical activity, pain while walking, and psychological activity) [35]. The recent Trans-Atlantic Inter-Society Consensus II update recommends the use of PLC in combination with physical training to improve the symptoms associated with PAD [35]. However, it has recently been reported that the long-term administration of PLC to patients with intermittent claudication did not result in a statistically significant improvement in peak treadmill performance or quality of life as compared with exercise alone [35]. The clinical effectiveness of L-carnitine in the treatment of other CVD entities is not well established [35].

 Chelation therapy is used to rid the body of excess or toxic metals (i.e., in lead poisoning). Some physicians and CAM practitioners have recommended EDTA chelation as a treatment for coronary heart disease (CHD) [1]. The bulk of evidence supporting EDTA chelation therapy is from case reports and case series, and the available randomized clinical trials, although underpowered, have seen no significant difference in direct or indirect measurements of disease severity and subjective measures of improvements [34]. The National Institutes of Health, including the National Heart, Lung, and Blood Institute and NCCAM, sponsored the Trial to Assess Chelation Therapy (TACT), the first large-scale, multicenter study designed to determine the safety and efficacy of EDTA chelation for patients with CHD [1]. Preliminary results of the trial were shared at the American Heart Association Scientific Sessions on November 4, 2012; however, the results will not be reported until they are published in the literature [1].

 The results of a systematic review indicate that supplement use is common in cardiac patients (26%–42%) and that the concomitant use of dietary supplements and prescription medication also appears to be frequent (16%–64%) [36]. These results are important for several reasons. Not only is the evidence regarding the efficacy of these products generally inconclusive or unfavorable, but also there is significant opportunity for danger in their use. Most patients believe that the government oversees the safety of CAM; however, the only requirement is for the manufacturer to send a copy of the product label to the FDA [37]. A new dietary supplement may be introduced and marketed rapidly despite containing new, experimental, or unregulated herbal ingredients, and many supplements contain ingredients or contaminants with adverse effects or interactions [37]. 

The general public regards biologically-based therapies as safe, natural, and as having fewer side effects than conventional medications (29%–60% of CAM users) [6, 37]. The lack of knowledge about herb-drug and herb-herb interactions and herb adverse effects by patients and health care providers is also problematic. A recent review determined that cardiovascular patients consumed on average seven prescribed medications and two herbal, vitamin, or mineral products daily [6]. One study identified 42 potential herb-drug interactions among these patients [6]. For instance, garlic may interact with aspirin, clopidogrel, warfarin, or heparinoids to increase bleeding risk [37]. Gingko biloba can increase hypoglycemia when taken with antidiabetes drugs and may increase bleeding when taken with aspirin or warfarin [37]. Ginseng also increases hypoglycemia with antidiabetes drugs, leads to falsely increased levels of digoxin, and decreases effectiveness of warfarin [37]. Hawthorn increases the effects of digoxin and increases coronary vasodilatory effects of calcium channel blockers or nitrates [37]. Echinacea increases QT interval when taken with amiodarone or ibutilide and increases the risk of hepatotoxic effects with statins, fibrates, or niacin [37]. St. John's wort decreases serum digoxin concentration, increases activity of clopidogrel, decreases warfarin effectiveness, decreases simvastatin effectiveness, and decreases the effectiveness of class IA and III antiarrhythmics [37]. Supplemental potassium was taken by 20% of patients in one study, which can result in adverse outcomes when used concomitantly with angiotensin converting enzyme inhibitors, aldosterone receptor antagonists, or angiotensin receptor blockers [6, 35]. One study demonstrated that 64% of the patients with a diagnosis of atrial fibrillation, CHF, or ischemic heart disease attending a cardiovascular clinic reported concomitant use of CAM and prescription drugs—58% took supplements that had potential interactions with warfarin, amiodarone, sotalol, or digoxin [37]. There are innumerable other interactions and side effects that must be taken into consideration when using biologically-based therapies.

### 2.2. Mind-Body Therapies

 The mind-body therapies (MBT) include anthroposophical medicine, autogenic training, biofeedback, bioresonance, cognitive-behavioral therapies, deep-breathing exercises, group support, hypnosis, imagery, meditation, prayer, relaxation, Qigong, tai chi, yoga, and shiatsu [3]. One review reported on the prevalence of MBT usage, which ranged from 2% to 57%, with deep breathing and meditation representing the most common therapies in the category [6]. In contrast to the complex and controversial body of research surrounding biologically-based therapies, there is a growing body of research suggesting that MBT are relatively safe and may have measurable benefits for cardiovascular health [5]. Cardiac patients used MBT most commonly for stress, emotional health, and general wellness—indeed, this use is supported by an established body of research on psychosocial support, stress management, and coping skills in cardiac rehabilitation and the influence of stress hormones, cortisol, and the hypothalamic-pituitary-adrenal (HPA) axis as mediators of cardiac risk [5]. Thus, the use of MBT for this purpose has become more widely accepted [5]. In fact, a systematic review suggested that MBT were cost-effective in patients with recent cardiac events and after cardiac surgery [5].

 Relaxation techniques include practices such as progressive relaxation, guided imagery, biofeedback, self-hypnosis, and deep-breathing exercises [1]. The goal of these techniques is to consciously produce the body's natural relaxation response, characterized by slower breathing, lower blood pressure and oxygen consumption, and a feeling of calm and well-being [1]. A 2008 Cochrane review found that interventions to promote relaxation were associated with a small, but statistically significant, reduction in both systolic blood pressure (5.5 mm Hg) and diastolic blood pressure (3.5 mm Hg) [38]. However, when relaxation was compared with sham therapy, the mean reductions in blood pressure were smaller and not statistically significant [38]. The review noted that in light of the poor methodological quality of the included studies, it is difficult to draw any definitive conclusions regarding the efficacy of relaxation techniques for primary hypertension or for reducing morbidity (myocardial infarctions and stroke) and mortality [38]. A 2008 double-blind, randomized trial comparing relaxation versus lifestyle modification found that both groups had similar reductions in systolic blood pressure; however, significantly more participants in the relaxation response group eliminated an antihypertensive medication while maintaining adequate blood pressure control [39]. Although more studies are needed regarding the effect of relaxation on heart disease, one observational study did find that combining relaxation response training with cardiac rehabilitation resulted in significant reductions in blood pressure, decreases in blood lipid levels, and increases in psychological functioning [40].

 Meditation refers to a group of techniques such as mantra meditation, mindfulness meditation, transcendental meditation, and Zen Buddhist meditation [1]. There is evidence that meditation is associated with potentially beneficial health effects. For instance, a meta-analysis found that transcendental meditation resulted in a reduction of 4.7 mm Hg in systolic blood pressure and 3.2 mm Hg in diastolic blood pressure [41]. Another review article suggested that transcendental meditation may reduce blood pressure as well as other risk factors for CVD such as cholesterol, oxidized lipids, and smoking [42]. However, most clinical trials on meditation practices are generally characterized by poor methodological quality with significant threats to validity in every major quality domain assessed [43]. Thus, future research must be more rigorous before firm conclusions may be drawn.

 Yoga has many different styles, some more physically demanding than others. In general, practicing yoga, as well as other forms of regular exercise, leads to several cardiovascular benefits. Yoga typically causes increased heart rate during the act, but following prolonged training, a decrease occurs in exercise-induced heart rate [44]. One study that looked at the effects of yoga on heart rate and blood pressure in healthy men found that the men in the yoga group showed greater decreases in heart rate and blood pressure and greater aerobic performance after 3 months as compared to the control group (flexibility exercises and slow running) [44]. Numerous studies have also commented on positive findings regarding weight loss, control of blood glucose, control of blood lipids, reduction in number of anginal episodes in patients with advanced coronary artery disease, and improved general quality of life [44]. Some research indicates that there may be a difference between yoga and exercise. Different levels of intensity of exercise have been shown to affect the HPA axis response to acute stress differently—low-intensity exercise lowers cortisol levels and sympathetic stimulation, while intense exercise raises cortisol levels and stimulates the sympathetic nervous system, raising levels of epinephrine and norepinephrine [45]. Exactly how this influences cardiovascular morbidity and mortality requires further research.

 Tai chi, sometimes referred to as “moving meditation,” encompasses many styles, but all involve slow, relaxed, gentle movements [1]. A systematic review of the literature determined that in 22 of 26 studies, reductions in blood pressure (3–32 mm Hg systolic, 2–18 mm Hg diastolic) with tai chi were reported [46]. Another systematic review also concluded that tai chi appears to have physiological and psychosocial benefits and appears to be safe and effective in promoting balance control, flexibility, and cardiovascular fitness in older populations with chronic conditions [47]. However, limitations and biases exist in most studies analyzed; thus, drawing firm conclusions about the benefits reported is difficult [47]. A recent randomized clinical trial found that tai chi may improve quality of life, mood, and exercise self-efficacy in people with chronic heart failure, despite the absence of differential improvement in peak oxygen intake and 6-minute walk test compared with education only [48]. Given that tai chi practice is safe and has good rates of adherence, it may represent an important complement to standard medical care in the treatment of deconditioned patients with systolic heart failure [48]. Further research is needed to explore these possibilities.

### 2.3. Manipulative and Body-Based Therapies

 The manipulative and body-based therapies include acupressure, Alexander technique, Bowen technique, chiropractic manipulation, Feldenkrais method, massage, osteopathic manipulation, reflexology, Rolfing, Trager bodywork, and Tui na [3].

 Massage therapy encompasses many different techniques, such as Swedish massage, sports massage, deep tissue massage, and trigger point massage [1]. Our study found that deep tissue massage resulted in a systolic blood pressure reduction of 10.4 mm Hg, diastolic pressure reduction of 5.3 mm Hg, mean arterial pressure reduction of 7 mm Hg, and an average heart rate reduction of 10.8 beats per minute directly after the massage took place [49]. A review of the literature remarked that single treatment reductions in salivary cortisol and heart rate were consistently noted, but sustained reductions for these measures were not supported in the literature [50]. No change was seen in urinary catecholamines at any point [50]. More research on the long-term effects of repeated messages is necessary.

 Spinal manipulation, as found in chiropractic and osteopathic manipulation, has been reported to successfully treat hypertension [51]. A systematic literature review however found that there is a lack of low bias evidence to support the use of spinal manipulation therapy to treat hypertension, as statistically significant decreases in blood pressure were not observed in trials with low bias [51].

### 2.4. Whole Medical Systems

 The whole medical systems include acupuncture (as part of traditional Chinese medicine), Ayurveda, homeopathy, and naturopathy [3].

 Acupuncture is a therapeutic modality anchored in traditional Chinese medicine (which also includes Chinese herbal medicine, moxibustion, cupping, Chinese massage, mind-body therapies such as Qigong and tai chi, and dietary therapy) [1]. A systematic review and meta-analysis that statistically pooled 3 sham-controlled trials out of 11 studies found that systolic blood pressure change was not statistically significant (−5 mm Hg) and acupuncture only marginally reduced diastolic blood pressure by 3 mm Hg, but substantial heterogeneity was observed [52]. When given with antihypertensive medication, acupuncture significantly reduced systolic blood pressure (−8 mm Hg) and diastolic blood pressure (−4 mm Hg) with no heterogeneity detected [52]. Given the poor methodological quality and small sample sizes of most acupuncture trials, the notion that acupuncture may lower high blood pressure is inconclusive [52]. A systematic review and meta-analysis of 29 randomized controlled trials found that acupuncture was associated with a significant reduction of average body weight of 1.72 kg compared to control of lifestyle and a significant reduction of body weight of 1.56 kg compared to placebo or sham treatment [53]. Again, given the poor methodological quality of the trials reviewed, it is difficult to say that the evidence is fully convincing [53]. There is also some evidence that acupuncture may help to correct various metabolic disorders such as hyperglycemia and hyperlipidemia, but further rigorous investigation in this area is warranted [54].

 Most rigorous clinical trials and systematic analyses of the research on homeopathy have concluded that there is little evidence to support it as an effective treatment for any specific condition [1]. There are mixed results concerning the research for the efficacy of naturopathy, and there is little scientific evidence currently available on the overall effectiveness of this treatment system [1].

### 2.5. Energy Medicine

 Energy medicine includes healing touch, light therapy, magnetic therapy, millimeter wave therapy, Qigong, Reiki, and sound energy therapy [3]. This category is reportedly the least utilized and least studied of the CAM modalities [3]. 

 The biofield therapies of Reiki, therapeutic touch, and healing touch are known as “hand-mediated” therapies and are used to reduce pain and anxiety and to promote health through the direction of healing energy [55]. There are reports describing changes in the physiological parameters of heart rate, skin temperature, muscle tone, and skin conductance in response to biofield therapies [55]. Most reviews of the most commonly researched energy therapies conclude that more research is needed, despite potentially promising findings [56]. A 2007 review concluded that studies of biofield therapies can only suggest efficacy in reducing anxiety, improving muscle relaxation, aiding in stress reduction, relaxation, and sense of well-being, promoting wound healing, and reducing pain [56]. A 2010 randomized controlled study published in the Journal of the American College of Cardiology found in a study of immediate postacute coronary syndrome inpatients that reiki significantly increased vagal activity as measured by high-frequency heart rate variability compared with resting and music control conditions, with a decrease in negative and an increase in positive emotional states [57]. The magnitude of the effect on heart rate variability seen was similar to that of propranolol in the Beta Blocker Heart Attack Trial [57]. A randomized clinical trial measuring the efficacy of healing touch in coronary artery bypass surgery recovery found no significant decrease in the use of pain medication, antiemetic medication, or incidence of atrial fibrillation; however, significant differences were noted in anxiety scores and length of stay, and all healing touch patients showed a greater decrease in anxiety scores when compared to the visitor and control groups [58]. More rigorous research is needed to determine the physiologic mechanisms and long-term benefits of these therapies.

## 3. Conclusion

 CAM use among patients with CVD is prevalent, with biologically-based and mind-body therapies being the most commonly used treatment modalities. This review illustrates the necessity of both more rigorous research to determine the precise physiologic effects and long-term benefits on cardiovascular morbidity and mortality with CAM usage as well as more open lines of communication between patients and physicians regarding CAM use. Finally, it is hoped that both physicians and patients gain an appreciation of what the evidence does and does not say with respect to CAM use for CVD and take this into consideration when determining optimal treatment plans.
